# Supplementary material for: Number of antenatal care visits and associated factors among reproductive age women in Sub-Saharan Africa using recent demographic and health survey data from 2008–2019: A multilevel negative binomial regression model
Source: PLOS Glob Public Health. 2022 Dec 27;2(12):e0001180. doi: 10.1371/journal.pgph.0001180 (PMC10022079; doi:10.1371/journal.pgph.0001180)
Supplement: Attachment — Submitted filename: Response to Reviewers.docx [file pgph.0001180.s001.docx]

**Response letter to reviewers**

**Title:** Number of antenatal care visits and associated factors among reproductive age women in Sub-Saharan Africa using recent demographic and health survey data from 2008-2019: a multilevel negative binomial regression model

**Authors;**

Fetene Getnet Gebeyehu: ([feteneget27@gmail.com](mailto:feteneget27@gmail.com))

Bisrat Misganaw Geremew: ([[bisratuog@gmail.com](mailto:bisratuog@gmail.com)](mailto:ashenafitazebew1@gmail.com),and))

Aysheshim Kassahun Belew: ([aysheshim121@gmail.com](mailto:aysheshim121@gmail.com))

Melkamu Aderajew Zemene: [(melmahman3m@gmail.com](mailto:(melmahman3m@gmail.com))

We are grateful for the helpful feedback from the Reviewers that helped us to improve the quality of the manuscript. We also appreciate your acknowledgment of the fact that this is a very important study describing the number of antenatal care visits and associated factors among reproductive-age women in Sub-Saharan Africa. We carefully responded to all points and have modified the manuscript accordingly.

With regards!

Authors

**Manuscript Submission ID:** PGPH-D-22-00897

**Response to Editorial Board**

Thank you, editor, for the clear instructions and suggestions. We did this point-by-point response to your suggestions.

**Response to Reviewers**

**Reviewer #1**: This is a good paper. However, it needs more work, and I recommended the authors to do the following;
1. The whole discussion needs to be written and synthesized. I think there are so many repetitive details, spelling, and grammatical errors.

Response: Corrected accordingly.
2. Results need to be presented succinctly and clearly. There is so much repetition also noted

Response: Revised accordingly.
3. The authors also need to maintain a good academic tone, use academic words, and maintain coherence and clarity.

Response: We tried to revise the new manuscript.
4. The authors need to check that all abbreviations are well used and defined.

Response: Corrected.
5. Authors need to recheck all punctuations and correct prepositions as stated in the review draft.

Response: Corrected.

**Reviewer #2**: Title of the paper: Number of antenatal care visits and associated factors among reproductive age women in Sub-Saharan Africa using recent demographic and health survey data from 2008-2019: a multilevel negative binomial regression model

Reviewer comments:
Specific comments:
Lines 105-114. The authors state the following:
The source population was all reproductive-age women who gave birth five years preceding each respective survey in sub-Saharan Africa, whereas those in the selected Enumeration Areas (EAs) were the study population. The sample size was determined from the kids to recode file “KR file” from the standard DHS dataset of Sub-Saharan African countries with at least one survey from 2008 to 2019. DHS uses a two-stage stratified cluster sampling technique. In the first stage, a sample of EAs was selected independently from each stratum with proportional allocation stratified by residence (urban & rural). In the second stage, from the selected EAs, households were taken by systematic sampling technique (23).
Comment: This narrative only tells us how the DHS selected the samples from within each country. But does not tell us how this process weights the sample across the 35 countries in the five identified geo-regions of Africa. This is necessary to establish how the computations of the merged sample was undertaken.

Response: All Sub-Saharan Africa member countries which had DHS data in the study period were included. Regarding weighting datasets, we made weights for each country’s dataset before appending and going to further analysis. Of course, appending all the datasets and making weight also gives the same finding. Therefore, the data from countries in the five identified geo-regions of SSA were made to represent the actual population at the sub-national level. However, we used the geo-regions of Africa as a second-level factor in the multilevel analysis to identify which region had a higher/lower outcome of interest. This is the usual way of weighting datasets for studies in SSA

Lines 114-116: The authors report the weighted sample size. This is not appropriate while reporting. The analysis uses the weighted sample. However, while reporting the actual number of women included needs to be reported. But in lines 195-196 in the results section the authors mention” A total of 256,425 reproductive-age women who gave birth within five years preceding the survey were included’. The weighted sample is also listed as 256, 425. The authors need to clarify this.

Response: Thank you very much. We revised the new manuscript as the final sample size was 257,924 (weighted sample 256, 425) women.
Lines 150-151. Sample weighting was done for each country before appending it to one dataset.
This is not an adequate way of weighing to obtain estimates for the DHS data sets of Africa. The authors need to explain or substantiate the process of using sub-national weights which will yield appropriate estimates for the nation across its strata are also equally applicable for the nation as a whole when it is merged into the collective African data set. This has not been described and it probably is not done. That renders the whole analysis invalid. It is likely that the model originally envisaged, that is the Poisson regression model because of this particular factor which would have demonstrated high variability because of the lower variation within a country and higher across the country thereby yielding a high sample variance (about two times that of the mean) thereby resulting in the choice of the negative binominal regression model. The authors seem to have overlooked this possibility.

Response: We checked both but we got the same findings. Firstly, sample weighting was done for each country before appending and further analysis. On the other hand, we appended all countries’ data together and then weighted it, but no difference.
Lines 193-195: The authors say: Variance inflation factor (VIF) was assessed to check for multi-linearity and the mean VIF for the fitted model was 1.86. Finally, a model comparison was done using the deviance test, and the model with the lowest deviance was selected as the best-fitted model.
Comment: The VIF shown is only for the last model that the authors present. However, each of the regressions in the exercise carries considerable collinearity that the authors do not check there by rendering the exercise inadequate.

Response: We reported the variance inflation factor (VIF) of the final model for it was the best-fitted model. However, we have checked collinearity from each model we fitted and it was not a problem. And reporting VIF is not necessary while using STATA as statistical software. STATA is a promising software that suggests variables that had collinearity in a model.
Overall comment:
The analysis is not appropriate as it has not described the process of weighing used to merge the African data set with appropriate weights. Further, the selection of variables within each model has not been justified using the VIF- where several variables could be conceptually associated, particularly maternal education, wealth index and media usage in model 2.
Given these limitations in analysis, and the need for computing weights for the continental analysis envisaged, this paper does not yield valid results for interpretation. I will not be able to recommend it for publication in its present form.

Response: As we replied above, there is no difference between weighting individual country data and appending OR appending the whole country’s data and weighting. Regarding collinearity, one advantage of using STATA software is that it suggests the variables if collinearity is the problem on the output window. Moreover, we doubled check even the VIF from the four models we fitted but we reported the final model for it was the final best-fitted model.

**Reviewer #3**: The analysis in the paper has used excellent analytical tool with proper justification of the method but not specified the research questions and theoretical conceptual model. As a result, they may not be clear why to analyze ANC service utilization as a count variable and what maybe it' programmatic implications.

Response: The nature of the outcome variable “number of ANC Visits” is a count data. Therefore, information will be lost while categorizing women's different numbers of ANC visits in the same group and fitting logistic regression. In this case, studies from count data analysis will give more reliable evidence than logistic regression.
Analysis using multilevel Negative Binomial distribution has not incorporated variation in model paraments under fixed effect across different social groups and also the implicit variability across sub-Saharan African countries have not been explored adequately. Thus, the paper may be accepted for publication is the authors revise it in the context of these.

Response: The present study tried to show that there are regional variations in the outcome variable across the different regions of SSA. The different regions of Africa have different levels of health service accessibility and overall service utilization. Thus, women from geographic areas with better health facilities and health care providers, as well as easy access to transportation, have a higher number of visits than their counterparts.

**Responses to comments attached to the manuscript:**

Line 23: Write in full form

Response: corrected

Line 43: Does this mean “planned pregnancy”?

Response: we can say planned pregnancy if wanted pregnancy confuses readers.

Line 56-58: This sentence needs to be re written for clarity

Response: It is revised.

Line 96-98: needs paraphrasing and grammar check

Response: Corrected.

Line 106: Reference?

Response: Reference inserted.

Line 132: write in full

Response: Corrected.

Line 191: Increase what?

Response: Increased ANC visits and it is corrected.

Line 195: Does this needs to be capitalized?

Response: Corrected.

Line 204-344: spacing, comma, spelling, grammar, plurality

Response: all these issues are resolved.

Line 278: The whole discussion needs to be written and synthesize. I think, there is so much repetitive details, spelling and grammatical errors. The also authors need to maintain good academic tone.

Response: we have revised the new manuscript accordingly. Thank you very much.

Line 304: Write in full

Response: Corrected.

Line 321-325: Please, paraphrase this sentence. It is too chucky to read and make sense of it. If possible, split the sentence into two. Use more academic words

Response: Corrected

Line 327-329: what does this mean? Paraphrase this

Response: Corrected.

Line 334: Are you talking about parity or multiple gestation?

Response: It is about either being single parity or multiparity. It is revised.

Line 341-345: Check this sentence. It needs a comma. Also, it is very chunky

Response: Corrected.

Line 346-349: Can you synthesize and combine the two sentences into one. Seemed to be very repetitive

Response: corrected in the revised manuscript.

Line 353-359: This paragraph has multiple errors, typos and needs synthesis and rewording. Lots of repetitive details

Response: it is revised.

Line 360-366: Need to use more academic words. This paragraph is too chunky and with so many repetitive details. It needs to be reworded, synthesized to be precise and clear.

Response: It is revised.

Line 367-372: Again, this paragraph is too chunky and with so many repetitive details. It needs to be reworded, synthesized to be precise and clear.

Response: It is revised.

Line 373-378: Not academic way of writing. Again, this paragraph is too chunky and with so many repetitive details and spelling errors. It needs to be reworded, synthesized to be precise and clear.

Response: It is revised.

Line 391: You have already defined World Health Organization in the previous part of the body. Check your use of abbreviations throughout the documents.

Response: Corrected.

Line 420: Edit the reference

Response: Edited.
