# Supplementary material for: Number of antenatal care visits and associated factors among reproductive age women in Sub-Saharan Africa using recent demographic and health survey data from 2008–2019: A multilevel negative binomial regression model
Source: PLOS Glob Public Health. 2022 Dec 27;2(12):e0001180. doi: 10.1371/journal.pgph.0001180 (PMC10022079; doi:10.1371/journal.pgph.0001180)
Supplement: Attachment — Submitted filename: Response to Reviewers.docx [file pgph.0001180.s002.docx]

**Response letter to reviewers**

**Title:** Number of antenatal care visits and associated factors among reproductive age women in Sub-Saharan Africa using recent demographic and health survey data from 2008-2019: a multilevel negative binomial regression model

**Authors;**

Fetene Getnet Gebeyehu: ([feteneget27@gmail.com](mailto:feteneget27@gmail.com))

Bisrat Misganaw Geremew: ([bisratuog@gmail.com](mailto:bisratuog@gmail.com))

Aysheshim Kassahun Belew: ([aysheshim121@gmail.com](mailto:aysheshim121@gmail.com))

Melkamu Aderajew Zemene: [(melmahman3m@gmail.com](mailto:(melmahman3m@gmail.com))

We are grateful for the helpful additional comments from the Editors that helped us to improve the quality of the manuscript. We also appreciate your acknowledgment of the fact that this is a very important study describing the number of antenatal care visits and associated factors among reproductive-age women in Sub-Saharan Africa. We carefully responded to all points and have modified the manuscript accordingly.

With regards!

Authors

**Manuscript Submission ID:** PGPH-D-22-00897

**Response to Editors**

The language in submitted articles must be clear, correct, and unambiguous. We recommend that authors seek independent editorial help before submitting a revision.

Response: Thank you, editor, for the clear comments and suggestions. We have given the paper to an independent editor and the language issue is improved.

Sufficient details were not provided on the sample size calculation and sampling procedure to enable the replication of your study. Please provide sufficient detail to allow reviewers to evaluate whether the conclusions of the study presented are generalizable to the wider population.

Response: The sample size was determined from the kids to recode file “KR file” from the standard DHS dataset of Sub-Saharan African countries with at least one survey from 2008 to 2019. The final sample size was 257,924 (weighted 256,425) women.

The following table shows how we determined the sample size. However, we assumed incorporating it in the manuscript might not be as such important.

Table: Sample size estimation to study the number of antenatal care visits and associated factors among reproductive-age women in sub-Saharan Africa using Demographic and Health Survey data from 2008 to 2019.

| **Sub-Saharan African Countries with Recent DHS report from 2008 to 2019** | | | | | |
| --- | --- | --- | --- | --- | --- |
|  |  |  |  | **Sample size** | |
| **Regions** | **Country** |  | **DHS year** | **Un weighted** | **Weighted** |
| East Africa  countries | Burundi |  | 2016/17 | 8660 | 8941 |
|  | Comoros |  | 2012 | 2011 | 2060 |
|  | Ethiopia |  | 2016 | 7193 | 7590 |
|  | Kenya  Madagascar |  | 2014  2008/09 | 14945  8546 | 14439  8641 |
|  | Malawi |  | 2015/16 | 13448 | 13515 |
|  | Mozambique |  | 2011 | 7623 | 7874 |
|  | Rwanda |  | 2014 | 5955 | 6060 |
|  | Tanzania |  | 2014/15 | 7050 | 7078 |
|  | Uganda |  | 2016 | 10263 | 10152 |
|  | Zambia |  | 2018 | 7372 | 7325 |
|  | Zimbabwe |  | 2015 | 4833 | 4988 |
|  | Angola |  | 2015/16 | 8947 | 8495 |
|  | Cameroon |  | 2018 | 6463 | 6613 |
| Central Africa countries | Chad |  | 2014/15 | 11081 | 11119 |
|  | Democratic Republic of the Congo |  | 2013/14 | 11288 | 11061 |
|  | Republic of the Congo |  | 2011/12 | 6458 | 5876 |
|  | Gabo  Sao tome & Principe |  | 2012  2008/09 | 4139  1431 | 3695  1363 |
|  | Benin |  | 2017/18 | 8994 | 9030 |
|  | Burkina Faso |  | 2010 | 10361 | 10484 |
|  | Ivory Coast |  | 2011/12 | 5425 | 5237 |
|  | Gambia |  | 2013 | 5385 | 5305 |
|  | Ghana |  | 2014 | 4294 | 4142 |
| West Africa  countries | Guinea |  | 2018 | 5530 | 5487 |
|  | Liberia |  | 2019/20 | 4267 | 4026 |
|  | Mali |  | 2018 | 6368 | 6623 |
|  | Niger |  | 2012 | 7678 | 8002 |
|  | Nigeria  Senegal  Sierra Leone  Togo |  | 2018  2010/11  2019  2013/14 | 21792  8147  7377  5016 | 21911  7678  7326  4858 |
| Southern Africa countries | Lesotho  Namibia  South Africa |  | 2014  2013  2016 | 2596  3952  3036 | 2575  3819  3036 |
|  |  |  |  |  |  |
|  |  |  |  |  |  |
|  | **Total sample size** | | | **257,924** | **256,425** |

The sampling technique applied for this study was a two‐stage stratified cluster sampling technique. In the first stage, a stratified sample of EAs was selected with probability proportional to the size and with independent selection in each sampling stratum. In the second stage, a fixed number of households were selected by equal probability systematic sampling in the selected EAs. The details are also available from the full DHS report and it is cited.

What items were considered when calculation wealth index, pls list-out.

Response: The current study was based on a secondary data analysis. We used wealth index as a categorical variable since DHS reports it from a Principal Component Analysis approach. DHS uniformly uses the following list of items:

Why Negative binomial distribution used? Pls provide sufficient details on selecting Negative binomial distribution with respect of this manuscript as authors discussed the Poisson regression model as well.

Response: For a count data analysis like a number of ANC visits, Poisson Regression is the first choice as far as the assumptions are fulfilled. However, the assumption of the Poisson regression failed; (i.e the sample variance exceeds the sample mean). this implies there was over-dispersion. In this case, a negative binomial regression model is the preferred one to handle over-dispersion and we used that as it was clearly stated in the manuscript.

What is the basis of selecting a p-value ≤ 0.2 as candidates for the final model.

Response:  It was a minor typing error. It is now corrected to p-value ≤ 0.25.

Thank you!!!

**Response to reviewers**

In this round we haven’t received new comments from any of the reviewers. Please refer to the previous round “Response to Reviewers” file. We are writing this because the same comments were sent.
